# Assessment of the informed consent process in the provision of dental care in Mulago hospital, Uganda

**DOI:** 10.1186/s12903-022-02550-2

**Published:** 2022-11-16

**Authors:** David Nono, Edward Mapley, Charles Mugisha Rwenyonyi, Isaac Okullo

**Affiliations:** 1 Cardiff School of Health Sciences, Cardiff Metropolitan UniversityLlandaff Campus, Cardiff, CF5 2YB UK; 2 School of Dentistry, College of Health Sciences, Makerere University, P.O.BOX 7072 Kampala, Uganda

**Keywords:** Comprehension, Dental patients, Dental practitioners, Informed consent practices

## Abstract

**Background:**

Informed consent is grounded in the principle of autonomy and represents patients’ right to participate in clinical decisions regarding their treatment. It is equally an ethical and legal requirement in dental care. The dental practitioner must offer appropriate information about all aspects of the treatment and ensure that a patient understands and makes an informed decision. There is limited literature on informed consent for dental care in Uganda. This study assessed patients’ comprehension of the informed consent process and dental practitioners’ practices in obtaining informed consent.

**Methods:**

This was a cross-sectional descriptive study conducted in the Dental Outpatient Department of Mulago Hospital. Two separate questionnaires were employed to collect data from dental patients and dental practitioners, respectively. Data were entered into Epi-data, coded, and imported into STATA 14 for statistical analysis.

**Results:**

Overall, the level of patients’ comprehension of the informed consent process was 91.1%, with 96.3% who felt the dental practitioners satisfactorily explained to them the treatment received and, 65.1% understood very well the information given to them. About 93.5% of the patients confessed that they were given other options of treatment while 98.5% consented before the dental practitioners started treatment.

Most dental practitioners 94.7% followed good clinical practices in obtaining informed consent and 98.7% gave information before initiation of treatment while 85.3% obtained consent from patients before starting any procedures. However, only 5.3% of the dental practitioners obtained written informed consent from patients.

**Conclusion:**

There is a need to devise ways of improving patients’ understanding of the treatment information given to them to support them make better and informed decisions regarding their care. Dental practitioners need to put more emphasis on the use of written consent in dental care because documentation helps in providing accountability and protects dentists from medical litigation in case the patients were to sue them for any treatment-related complications.

## Introduction

Informed consent is an important aspect of clinical care ethics [1]. It is grounded in the principle of autonomy [2] and symbolizes patients’ right to participate in clinical decisions regarding their treatment [3]. Informed consent to treatment is equally an important aspect of the proper provision of dental care and it is a legal requirement for every dental procedure [3].

The need to obtain informed consent has been acknowledged in different health care specialties given the associated complications, invasiveness of the procedures, and the costs involved [4]. There is increasing support for the doctrine of informed decision-making as a way of ensuring patient autonomy in healthcare; the need to assess, protect and enhance patients’ capacity to freely make decisions about their health care instead of clinicians making for them decisions [2]. With the increasing technological advancement in the medical field leading to easy access to education and information, there is a need to encourage greater personal independence and the respect of patients’ rights in decision making [5].

The dental practice is guided by the same principles that regulate the doctor-patient relationship, hence the shared decision-making process is a requirement of good dental care practice [6]. Therefore, in a dentist-patient relationship, the dentist must offer appropriate and easily understood information about all aspects of the treatment as well as commitments after treatment [6].

In Uganda, informed consent is a legal requirement; in terms of legal instruments, according to chapter four of the constitution of Uganda, the rights and freedoms of the individual shall be respected, upheld and promoted by all organs, agencies of Government and by all persons. Furthermore, the Uganda Medical and Dental Practitioner Council (UMDPC) established in 1913 by the Act of Parliament published the Code of Professional Ethics (2013), and section 7 states that a practitioner shall not conduct any intervention or treatment without consent.

According to the Uganda Ministry of Health Patient’s Charter, Article 10 provides that each patient has the right to be given adequate and accurate information about the nature of one’s illness, diagnostic procedures, and the proposed treatment for one to make an informed decision. The information should be communicated to the patient at the earliest possible stage in a way that he or she is expected to understand so as to make a free informed and independent choice.

In Brazil, a study on the use of informed consent forms, 95.5% of the dentists provided verbal explanations on the treatment plan to their patients, 14.5% used informed consent forms every time in dental practice while 48% used the forms occasionally and only in special cases and 37% did not use informed consent forms in clinical care [7]. In a similar study in Nigeria, 61.6% of dental practitioners obtained written informed consent from their patients, majority (70.1%) of which were for surgical procedures [8]. In Uganda, Ochieng et al. reported that 48.8% of medical surgeons received informed consent from patients before performing surgery while 88.6% reported obtaining informed consent at the last surgical operation [9]. However, there is no published study that specifically looked at informed consent in general dental care in Uganda. The purpose of the present study was to investigate dental patients’ comprehension during informed consent process and dental care practitioner’s practices in obtaining informed consent in Mulago Hospital, Uganda.

## Material and methods

### Research methods and designs

This was a cross-sectional quantitative study; data were collected through a survey.

### Study site

The study was conducted among patients and dental care practitioners in the dental clinic in Mulago Hospital, a national referral and teaching facility located in Kampala City, the capital of Uganda which has a capacity of 2000 beds and a number of specialized clinics including dental clinic which attends to approximately 100 outpatients per day (personal communication, Medical Records Registrar). Mulago Hospital was chosen as a study site because it is a government facility, located in central Uganda, and is accessible to a lot of people. It serves very many patients from the Kampala Metropolitan as well as patients from other parts of Uganda because it recruits highly trained personnel.

### Selection of study participants

A total of 324 patients aged 18 years and above who received treatment at the dental clinic and gave informed consent were recruited into the study using a consecutive sampling procedure. In addition, a total of 75 dental care practitioners (including dental surgeons, dental interns, and public health dental officers) who consented to participate in the study were purposively selected using census. The sample size for patients and dental practitioners were obtained using the Krejcie and Morgan (1970), based on the sampling frame for patients (2050) and dental practitioners (93) respectively [10].

### Exclusion criteria

Patients who were very sick and unable to speak and those below 18 years of age were excluded from the study. Dental care practitioners who were absent during the data collection period or those unwilling to consent were excluded from the study.

### Data collection procedure

Standardized questionnaires were developed and pretested for errors and clarity before data collection. A structured questionnaire was administered by a research assistant to patients to obtain information about *their comprehension of the informed consent process*.

The level of patients’ comprehension of the informed consent process was measured using a Likert scale which ranged from 1-to 5, where 1 represented strongly disagree and 5 represented strongly agree. The dental practitioners (*n =* 75) were given a self-administered questionnaire to *assess their practices in obtaining informed consent from patients.*

### Data management and analysis

The collected data were entered into Epi data version 3.1 software, cleaned, and double checked for errors, and completeness. They were then exported to STATA version 14 software for analysis. Descriptive statistics in form of frequencies and proportions of the participants were used to determine the overall level of patients’ comprehension of the informed consent process. Chi-square statistics were used to determine the association between independent and dependent variables. *P*-value< 0.05 was considered statistically significant.

### Ethical consideration

Ethical approval was obtained from Mulago Hospital Research and Ethics Committee (Reference Number MHREC 2099) as well Cardiff School of Sports and Health Sciences Ethics Committee (Reference Number PGT-4393). Permission to carry out the study was obtained from the administration of Mulago Hospital.

Written informed consent was obtained from all participants who took part in the study. The purpose of the study was explained to the participants and their participation was voluntary and agreeing to participate did not waiver their rights in any way in accordance with the Helsinki Declaration [11]. All the data collected were kept securely in a cabinet under lock and key and only accessible to the investigator.

## Results

A total of 324 patients took part in the study. The majority of the participants were Moslems 93 (28.7%) while 312 (90.7%) had previously received dental treatment as shown in Tables 1 and 2 respectively.

**Table 1:** The frequency distribution of patients’ socio-demographic characteristics (*n =* 324)

Categories	Frequency (n)	Percentage (%)
**AGE IN YEARS**		
18–29	159	49.1
30–39	58	17.9
40–49	52	16.1
50 and above	55	16.9
**GENDER**		
Male	169	52.2
Female	155	47.8
**MARITAL STATUS**		
Single	127	39.2
Married	177	54.6
Divorced	3	0.9
Widow/Widower	17	5.3
**LEVEL OF EDUCATION**		
Informal education	12	3.7
Primary	28	8.6
O-Level	58	17.9
A-Level	87	26.8
Tertiary	139	42.9
**RELIGION**		
Catholic	83	25.6
Anglican	74	22.8
Seventh-Day Adventist	24	7.4
Pentecostal	49	15.1
Muslim	93	28.7

**Table 2:** The frequency distribution of patients’ responses on consent (*n =* 324)

Category	Frequency	Percentage
**Level of comprehension**		
High	295	91.1
Low	29	8.9
**Have you ever received dental treatment before?**		
YES	294	90.74
NO	30	9.26
**Do you feel the dental practitioner explained the treatment he/she carried out?**		
YES	312	96.30
NO	12	3.70
**If yes, how well did you understand the explanation?**		
I did not understand	4	1.28
I somehow understood	11	3.53
I understood	94	30.13
I understood very well	203	65.06
**Were you told of the other options of treatment?**		
YES	303	93.52
NO	21	6.48
**Did you give the dental practitioners permission for the treatment done to you?**		
YES	319	98.46
NO	5	1.54
**If yes, was it verbal, written or both?**		
Verbal	299	93.73
Written	1	0.31
Both	19	5.96
**Did you ask any questions?**		
YES	308	95.06
NO	16	4.94
**Do you know the name of the dental practitioner who gave you the treatment?**		
YES	290	89.51
NO	13	4.01
**If no, why?**		
Have forgotten	10	3.09
Was not told	11	3.4

Patients’ comprehension of the informed consent processes was measured using a Likert scale. The respondents who had indicated strongly agree, Agree and Neutral were constituted into a composite variable as High level of comprehension while those who responded as Disagree and strongly disagree were considered as low level of comprehension. Overall, 295 (91.1%) had a high level of comprehension of the informed consent process (Table 2). Most respondents 312 (96.3%) agreed that the attending dentist explained to them the treatment they were going to receive and 303 (93.5%) explained that they were given other options of treatment. About 319 (98.5%) of the respondents consented before receiving treatment.

Out of the patients that consented, 299 (93.7%) gave verbal consent, 1 (0.3%) gave written consent while 19 (5.96%) gave both verbal and written consent. About 308 (95.1%) asked questions about the treatment they were going to receive (Table 2).

### Bivariate analysis

In bivariate analysis, there was no independent variable that was statistically significantly associated with the patients’ level of comprehension of the informed consent process. This implies that no characteristic significantly influenced the participants’ level of comprehension as shown in Table 3.

**Table 3:** The frequency distribution of patients according to association of patients’ socio-demographic characteristics with level of comprehension (*n =* 324)

Categories	Comprehension		Chi-Square	*P*-value
	YES n (%)	NO n (%)		
**Age in years**				0.5
18–29	146 (91.8)	13 (8.2)		
30–39	50 (86.2)	8 (13.8)	2.43	
40–49	49 (94.2)	3 (5.8)		
50 and above	50 (91.9)	5 (9.1)		
**Sex**				6
Male	152 (89.9)	17 (10.6)	0.532	
Female	143 (92.3)	12 (7.7)		
**Marital status**				
Single	114 (89.8)	13 (10.2)		0.9
Married	162 (91.5)	15 (8.5)	0.798	
Divorced	3 (100)	0 (0.00)		
Widowed	16 (94.1)	1 (5.9)		
**Religion**				0.09
Anglican	70 (94.6)	4 (5.4)		
Catholic	11 83 (86.7)	11 (13.3)		
Seventh-day Adventist	0 24 (100)	0 (0.00)	9.468	
Pentecostal	41 (83.7)	8 (16.3)		
Muslim	8 87 (93.5)	6 (6.5)		
**Level of Education**				0.7
informal education	12 (100)	0 (0.00)		
Primary	25 (89.3)	3 (10.7)	2.252	
O-level	54 (93.1)	4 (6.9)		
A-level	80 (91.9)	7 (8.1)		
Tertiary	124 (89.2)	15 (10.8)		
**Occupation**				0.8
Unemployed	84 (92.3)	7 (7.7)		
Subsistence farmer	16 (94.1)	1 (5.9)	0.883	
Self-employment	108 (89.3)	13 (10.7)		
Formal employment	87 (91.6)	8 (8.4)		

### Socio-demographic characteristics of dental practitioners

Most of the practitioners 52 (69.3%) had practiced for 1–10 years and majority 25 (33.3%) were Catholics (Table 4).

**Table 4:** The frequency distribution of dental practitioners according to socio-demographic characteristics (*n =* 75)

Variables	Frequency (n)	Percentage (%)
**AGE IN YEARS**		
1–29	26	34.7
30–39	36	48.0
40–49	13	17.3
**GENDER**		
Male	50	66.7
Female	24	32.0
No response	1	1.3
**MARITAL STATUS**		
Single	29	38.7
Married	46	61.3
**QUALIFICATION**		
Public Health Dental Officer	38	50.7
Bachelor of Dental Surgery	36	48.0
Master of Dentistry	1	1.3
**Years of dental practice**		
0–10	52	69.3
11–20	22	29.4
21–30	1	1.3
**RELIGION**		
Catholic	25	33.3
Anglican	22	29.3
Seventh-Day Adventist	3	4.0
Pentecostal	22	29.3
Muslim	3	4.0

### Informed consent process of dental practitioners

Most of the dental practitioners 71 (94.7%) followed good, informed consent process (informing the patient of the available treatment options before initiating treatment, documenting findings and treatment to be followed). About 74 (98.7%) provided information before initiating treatment and 64 (85.3%) obtained consent (Table 5).

**Table 5:** The frequency distribution of dental practitioners according to informed consent process (*n =* 75)

Patient characteristics	Dental practitioners’ practice	
	YES n (%)	NO n (%)
**Clinical practices followed by dental practitioners**	71 (94.7)	4 (5.3)
**Information is given before initiation of treatment**	74 (98.7)	1 (1.3)
**Do you take consent from patients before starting any procedures?**	64 (85.3)	11 (14.7)
I do administer written informed consent	4 (5.3)	71 (94.7)
I do verbal consent	57 (80.3)	14 (19.7)
I do both verbal and written consent	14 (19.7)	57 (80.3)
**Type of consent obtained from Illiterate patients**		
Verbal consent	68 (90.6)	7 (9.4)
Patient’s thumbprint	30 (40)	45 (60)
Signature next of kin	18 (24)	57 (76)
Verbal consent and thumbprint	34 (45.3)	41 (54.7)
**If a patient asks to take a copy of the consent form, did you provide a copy?**		
Provide the form willingly	64 (85.3)	11 (14.7)
Ask for a reason before giving a form	16 (21.3)	59 (78.7)
Refuse to give the form	70 (93.3)	5 (6.7)
**Do you obtain parents’ informed consent when treating their children?**		
Yes	51 (68.0)	24 (32.0)
Always	21 (28.0)	54 (72.0)
No	2 (2.7)	73 (97.3)
In definite cases only	1 (1.3)	74 (98.7)

Dental practitioners who obtained written informed consent were 4 (5.3%), 57 (80.3%) obtained verbal informed consent while 14 (19.7%) obtained verbal and written consent.

For illiterate patients, 68 (90.6%) of the practitioners obtained verbal informed consent while 64 (85.3%) would willingly give the form to their patients and 51 (68.0%) practitioners sought informed consent from parents before treating their children (Table 5).

## Discussion

In the present study, the overall level of patients’ comprehension of the informed consent process was 91.1% which is comparable to 96% reported in an earlier study [12]. This high value could be due to patient’s awareness of the “right to know” conditions before treatment and ability to search about their ailment on the internet, improved communication techniques, provision of adequate time to the patients by the dentists to explain treatments options and quality of the information provided to patients by the dentists.

 Bivariate analysis using Chi-square test showed that no factor was significantly associated with the patients’ comprehension of the informed consent process, which is in contrast with findings in other studies [13, 14], particularly, where patients’ religious beliefs affected liberty and decision as to whether to accept or decline a recommended medical intervention. Not having a strong association between participant characteristics and level of comprehension implies there is a possibility that certain characteristics of the study population or confounders that were not controlled for influenced these results hence not being able to make a legit conclusion about the study results. However, this does not mean that the findings are null and void. Additionally, having no influencing factor means participating patients had no bias concerning treatment at the time of data collection.

Although it was not statistically significant, female patients had a higher level of comprehension compared to male counterparts (Table 3). This could be contributed to the fact that females have a higher prevalence of health seeking behavior [15]. Similarly, patients who had tertiary education had higher level of comprehension compared to their counterparts with a lower education (Table 3).

This is because education may help the recipient understand the language used and information delivered by the dental care provider [16]. It is imperative that how the information is explained to the patients should also vary depending on one’s level of education to enhance their understanding.

Generally, 94.7% of the dental practitioners were informing the patients of the available treatment options before initiating treatment, documenting findings and treatment to be followed. They followed good clinical practices in obtaining informed consent comparable to 97.4% reported in an Indian study [17]. This is also similar to findings by Kotrashetti in Belgaum City in India where 93.2% of the dentist discussed the various treatment modalities available at their clinic with their patients before starting treatment [18]. These findings show that most of the dental practitioners are complying with the ethical and legal requirements stated in the Uganda Medical and Dental Practitioners Council Code of Professional Ethics and the Uganda Ministry of Health Patients’ Charter. However, when it came to obtaining consent for treatment, only 5.3% obtained written consent from the patients. In a related study conducted in Nigeria, 61.6% of the dentists obtained written consent from their patients [8], while Kotrashetti and colleagues found that 63.6% of the dentist took written consent from the patients. About 80.3% of the dental practitioners got verbal consent from the patients (Table 5), which corroborates findings in a study [19] in Pakistan, but almost double the value (46.3%) reported in Bulgaria [5]. These studies showed that most of the dental practitioners are not taking written consent from patients. Article 10 of the Uganda Medical and Dental Practitioners Council Code of Professional Ethics states that consent may be given verbally, however it would be ideal to have the consent documented because if patients consent verbally and it is not documented, in case of injury resulting from a surgical procedure or instance, it may bring litigating issues yet if consent is documented, it can give the dental practitioners protection from medical litigation. The documented consent can serve as proof that the patient was informed about the possible risks of the treatment they were going to receive.

Medical litigation is on the rise in Uganda as patients in Uganda are becoming aware of information related to medical treatment as well as their rights through mass media and social media. With increase access to smartphones and internet coverage, and increased availability of legal services rendered by lawyers, medico-litigation is likely to increase [20]. Therefore, more efforts need to be done to create awareness on this oversight among practitioners both in Uganda and the world over.

In comparison of the data from patients and dental practitioners, majority of patients (96.3%) felt that the dental practitioners had explained to them about the kind of treatment they were going to be given. This seems to be relative level of consistency with what the dental practitioners reported, where 94.7% reported informing the patients of the available treatment options before initiating treatment, documenting findings and treatment to be followed. Regarding obtaining written consent, there is also some level of consistence with what was reported by the patients and dental practitioners, where 93.7% of patients reported providing verbal consent, while 80% of dental practitioners obtained verbal consent. It is important for patients to also demand to have their consent documented and where possible obtain a copy of the consent form. This helps them to keep a record of what they consented to for any future reference. Furthermore, 95% of the patients reported asking questions about the treatment they were going to receive, this could have possibly given the dental practitioners to provide more explanation to help the patients understand better and make an informed choice. This implies that communication between the patients and health care providers is crucial to ensuring that there is information sharing between the two parties and also helping the dentists to find the best way to deliver the information to the patients to enhance their understanding.

Good communication between the health practitioner and patient enables the patients to share important information necessary and it is essential for an accurate diagnosis of their condition, enables health practitioners have a better understanding of their patients’ treatment needs, in turn leading to a better treatment [21].

### Implications of the findings

The present study showed that most of the dental practitioners do not obtain written consent from patients, which can lead to possible medical litigation in case a patient is harmed while undergoing a procedure. Therefore, apart from verbal explanations from dental practitioners, there is a need to promote documentation of the consent process, availing patients with written information about a procedure can help them to read and internalize the information over and over again and also give them time to reflect, consult and make proper decisions that are well informed. At bivariate analysis, there was no independent variable that was statistically significantly associated with the patients’ level of comprehension of the informed consent process. Future research can be conducted on a different population or look at a different set of variables within the same population.

### Limitations

Considering that the study was conducted at a time when the country was in lockdown, due to the COVID 19 pandemic, it was not possible to observe the dentist-patient interaction during the consenting process, which may be prone to recall bias hence this could affect the generalizability of the findings.

### Further research

This was a quantitative study, which calls for a need to use qualitative methods to explore more about the dental practitioners’ experiences and perspectives of obtaining informed consent as well as patients’ experiences during the informed consent process.

## Conclusion/recommendation

Overall, the level of patients’ comprehension of the informed consent process was very good. The dental practitioners should put more effort into ensuring that this is maintained, by promoting communication with the patients and encouraging them to ask questions to ensure that all patients adequately understand the relevant information on the procedures that they are going to undergo.

Dental practitioners had good clinical practices in providing information before initiation of treatment, and got consent from patients before starting any procedures. However, most of the dental practitioners got verbal consent from their patients. The dental practitioners should be encouraged to embrace documentation of informed consent since written consent is a more recognized form of consent compared to verbal consent and can offer them protection in case they are sued by the patients.

## Data Availability

Data sources are available on request. The request can be sent to the corresponding author at nndvd45@gmail.com
